# Psychotherapy in India

**DOI:** 10.4103/0019-5545.69270

**Published:** 2010-01

**Authors:** L. S. S. Manickam

**Affiliations:** Department of Psychiatry, JSS University, JSS Medical College Hospital, Mysore - 570 004, India

**Keywords:** Psychotherapy, psychotherapy research, India

## Abstract

The articles that appeared in Indian Journal of Psychiatry were related to different areas of psychotherapy. Case reports dealt with a wide variety of cases. The review papers focused on the suitability of psychotherapy in the Indian context, different approaches in psychotherapy, psychotherapy training and supervision. Psychotherapy has been viewed very close to faith orientation. There were attempts to identify the indigenous concepts that are applicable to psychotherapy. Empirical studies are low in number. Concerted effort is needed to generate interest in psychotherapy, conduct more research on evidence-based therapies as well as on psychotherapeutic process variables.

## INTRODUCTION

Psychotherapy research has entered a new phase in the new century with more than 450 different therapies. From experiential and introspective narrations, it has taken a different turn with more evidence-based research appearing in the recent years in the Indian Journal of Psychiatry (IJP), spanning the period from 1959 to 2007.[1–3] Search for psychotherapy yielded 92 results in the IJP. On the other hand, search for ‘psychotherapy research’ showed 31 results which included case reports, editorials as well as book reviews. However, abstract search showed no results for the key word ‘psychotherapy research’. Research studies related to psychotherapy process variables like empathy did not come up while searching for psychotherapy.

The psychotherapy articles that appeared in the IJP can be considered as occurring in three different phases in relation to the theoretical allegiance it has taken - initial phase, middle phase and the current phase. In the initial phase there were more articles related to psychoanalytically-oriented concepts. In the middle phase the articles were related to different theoretical concepts-psycho dynamically oriented, humanistic and existential. In the current phase, there are articles that take more of an eclectic and integrative stance. However, all through these phases, one finds an attempt to integrate indigenous concepts in the application of psychotherapy - The one appearing as early as in 1959[1] to 2004.[4] Analysis of the nature of articles that came up in the search showed that it included those articles that mentioned the need for psychotherapy in different form of disorders too.[5,6]

## CASE REPORTS

The case reports that appeared showed a wide variety of cases. One of the earlier reports in 1958 was related to the successful treatment of a patient with schizophrenia using psychoanalytically-oriented psychotherapy.[7] Rao reported a case that was treated with Existential therapy in Anxiety Neurosis without medication.[8] The other reports have used psychotherapy as an adjunct in different disorders and the application of psychotherapy with in-patients with schizophrenia.[9] Supportive Psychotherapy was used as an adjunct to treat ‘factitious schizophrenia’[10] whereas insight-oriented psychotherapy was used as an adjunct to treat Dermatitis artefacta.[11] One of the case reports published was from Brazil wherein the psychopathology of flash backs in social anxiety disorder was discussed.[12] Nagaraja narrated how psychotherapy was effectively used in treating adolescents in Hysterical Twilight state.[13] Bassa analyzed the cases at child psychotherapy centers; however, there is no mention of psychotherapy among children.[14] He suggested the need for orientation of pediatricians towards psychogenesis of various disorders to help prevent ‘psychosomatic disorders’. In another case report, childhood sexual abuse was brought out in five adults during the process of supervised psychotherapy.[15] Bastani reported group psychotherapy with male genital exhibitionists conducted in US.[16]

## DIFFERENT APPROACHES IN PSYCHOTHERAPY: CROSS ROADS

Psychotherapists in the Indian context face various dilemmas. Even as early as in the 1950s, Nand acknowledged the ‘split’ of psychiatrists into biologically-oriented and analytically-oriented.[1] Later, many others later observed that psychotherapy, as practiced in the West, might be suitable only for those living in cosmopolitan cities of India and not for majority of the population.[17–24] It was felt that one has to not only use a tool that is ‘alien’ to the culture, but also the socio-cultural milieu, which at times appears contradictory to the basic tenets of psychotherapy. Surya and Jayaram pointed out that the Indian patients are more dependent unlike Western patients who look for integration of intra psychic processes; there is a tendency for dissociation between thinking, feeling and acting and may block the process of psychotherapy.[20] Neki discussed confidentiality and privacy in the Indian context and opined that these terms do not even exist in Indian Languages and, in the socio-cultural context; the concepts of privacy could severe people from interdependent society.[17] Therefore, he recommended family therapy or at least couple of sessions with the family members along with dyadic therapy in order to help the progress of the psychotherapy.

Verma raised objections to the applicability of the Western type of psychotherapy in India.[19] He pointed out seven distinct features of the Indian population, which may not help psychotherapy work in the Indian context in comparison to the western population. They are as follows:

1. Dependence/interdependence.
2. Lack of psychological sophistication.
3. Social distance between the doctor and the patient.
4. Religious belief in rebirth and fatalism.
5. Guilt attributed to misdeeds in past life.
6. Confidentiality.
7. Personal responsibility in decision making.

He also viewed that the history of psychotherapy in India shows that it differed from the West in the following lines:

1. It was not meant only for the sick.
2. The patient and the therapist cannot be considered equals and hence dyadic relationship is not possible.
3. The patient has to accept what the therapist considers as ‘truth’.
4. Everyone is not considered fit for psychotherapeutic relationship.

However, with globalization, increasing levels of education, higher sense of awareness on human rights and the wider use of electronic media even among the rural population, whether these observations stand today is a pertinent question. Varma and Ghosh,[23] in a study, of the practice of psychotherapy, on 32 Fellows of the Indian Psychiatric Society, found that short-term supportive therapy was used by majority of them. Some practiced other forms of psychotherapy including psychodrama.

On the other hand, Shamasunder held the view that psychotherapy can be effectively conducted on the Indian population.[24–27] Rao practiced existential psychotherapy and substantiated that the existential philosophy is not alien to ‘Eastern’ culture and can be used effectively.[7] He also argued that those who took a stance against the suitability of ‘Western Psychotherapy’ in Indian culture were focusing their arguments based on the psychoanalysis and not on other forms of psychotherapy of Western origin and emphasized that existential philosophy is very much closer to the Indian philosophical psychology.

However, the need to give adequate importance to psychotherapy, not only in treating psychiatric disorders but also from a public health perspective, in developing countries is also highlighted.[28–31]

## REPORTS FROM ABROAD

Dreikers observed the usefulness of Adlerian psychotherapy in correcting faulty social values based on his experience in USA.[32] Cameron[33] wrote in 1961 based on his experience of practicing psychotherapy in Canada, “psychotherapy is undoubtedly the most widely used therapy within the field of psychiatry,” which may not go well with the situation in India. Errazquin, in his review, observed the usefulness psychotherapy in treating psychoses.[34] Lesse, who is trained in psychoanalytically-oriented psychotherapy reported on specially designed psychotherapeutic procedures used in combination with drugs, useful for out patients with different psychiatric disorders. He advocated psychotherapy to be used intentionally secondary to the administration of drugs.[35] Stringham’s article provides an overview of psychotherapy.[36]

## FAITH ORIENTATION AND PSYCHOTHERAPY

Psychiatrists practicing psychotherapy in India have published the influence of religion as an essential ‘ingredient’ of psychotherapy from the very early days. Nand compared scientific psychotherapy with ‘Religious Psychotherapy’.[3] He observed ‘Shivite symbol’ as one fixed symbol for Indian culture. Based on the case records of Indian patients, of Hindu faith, he compared it with the phallic symbols represented at the temples at Banaras. While discussing ‘total psychoanalysis,’ he drew parallel between the symbols that Freud brought in from his religious and cultural traditions into psychoanalysis and explored its counter part in the Indian patients. While doing so, he suggested the need to strengthen religious therapy as well as ‘race therapy’ and explore the psychopharmacology of the change agents in psychotherapy. Hosseini described how Islamic principles could be used in psychotherapy.[37] Hoch too examined the way Pirs and Faquirs function as therapists and interpreted the indigenous concepts involved in their practices as ‘therapists’ that worked with illiterates.[38]

### Psychotherapy concepts from Indian philosophical psychology: Energy from ancient tradition?

One finds that as early as in 1961 there have been attempts to integrate indigenous concepts in the application of psychotherapy.[3,39] Nand has brought in the ‘Shivite’ symbols in psychoanalysis. Surya and Jayaram observed that the legend of Savitri provides the framework of psychotherapy.[20] Verma viewed psychotherapy as, the ‘interpersonal method of mitigating suffering’ and found its roots in the communication of Buddha; he also emphasized the use of concepts of Karma and Dharma in psychotherapy.[19] Neki used the concept of *Sahaja*.[18] He considers the healthy woman personality possessing- illumination, equipoise, spontaneity, freedom and harmony- a higher state of positive mental health as manifested through Sahaja. He also discusses the potential of various other Indian concepts including *nirvana*. One of his concepts is related to relationships in psychotherapy- *Guru-chela*, which he viewed as a therapeutic paradigm.[22] *Patanjali yoga* as a therapy had also been used.[40,41] Wig used the term Hanuman complex[4] and the mythology for helping the patients and the doctors understand about process of psychotherapy. The therapist quality of *Sahya* is another concept from Indian thought that has to be explored.[42]

### Psychotherapy training

There is an increased demand from psychotherapy trainers and trainees for better psychotherapy training procedures.[26,27] However, reports on psychotherapy training are very meager.[43,44] Rao[8] delineated five reasons for the neglect of psychotherapy training in India. He observed that:

1. Psychotherapy is highly subjective and individualistic.
2. Novelty of the psychological and philosophical concepts to the trainee.
3. Impracticability of Analysis of the trainee as required by some psychoanalytically oriented approaches.
4. Time required for training that extends beyond the training period; and
5. Lack of inclination among trainees.

The availability of trainers who are inclined, interested and committed to impart the psychotherapeutic skills is reiterated by many psychiatrists.[8,24,43] The number of training centers that devote time on psychotherapy is also few. Shamasundar opined that in the absence of a wide network of specialist psychotherapy services in the country, it is imperative for the general psychiatrist to have a ‘working knowledge of psychotherapy’. Psychotherapy training needs to form an essential part of psychiatric training.[24] He suggests the inclusion of psychotherapy training as a desirable component of all medical postgraduate training.

### Supervision in psychotherapy

Psychotherapy training requires a strict supervision of the work of the young therapist by an experienced therapist. The young therapist in our country, many a times, is not giver adequate supervision and the final product is a therapist without adequate skills.[26] Shamasunder appealed for active, supervised training in psychotherapy for junior and trainee psychiatrists.[26] The supervising task becomes more cumbersome due to multilingual nature of the patients, trainees and trainers. Tharyan, in an experiment, showed that group supervision is feasible and acceptable in a general hospital psychiatry set up in India though it cannot replace individual supervision.[43]

### Psychotherapy research

Dhairyam made an early call for psychotherapy research.[45] However, there is paucity of literature related to psychotherapy process and outcome research undertaken in our country. More than two decades ago Neki suggested that research on process variables to be initiated, in order to develop a unique perspective on psychotherapy in our country that is congruent to our culture including that of *mauna*.[18] There appear to be no takers to the challenge and this could be due to various factors. One of them could be very few training centers of psychotherapy in our country. Even in the existing centers, lack of sophisticated gadgets required to conduct a well-designed control study might have prevented researchers from taking up studies related to psychotherapy variables.

### Case control studies on psychotherapy

Two of the earliest studies are those of Balakrishna *et al*.[40] and Vahia.[46,47] Balkrishna and his associates studied the effect of *Patanjali yoga* on ‘psycho neuroses’ and found it useful in stress induced psychological disorders. It showed better results than the drug treatment. Probably Vahia’s studies introducing *Patanjali yoga* into psychiatry research was a landmark that led many others to take up *yogic asanas* and related yogic concepts for empirical research in the field of psychiatry and health.[46,47] In another case-controlled study, Kumar and Thomas assessed the effectiveness of brief psychotherapy in a sample of patients with alcohol dependence.[4] They concluded that a combination of psychopharmacological treatment with appropriate psychosocial therapies that is focused on the specific problem of the patient might provide better outcome than either of the therapies given alone. However, it was not a blind study and hence the results obtained may have been confounded by other variables too.

### Psychotherapy process variables

Research on empathy was conducted on different professional groups, -psychiatrists, clinical psychologists, psychiatric social workers and lay counselors.[48,49] Though the study was conducted using simulated client, the participants of the study perceived the simulated client similar to that of a real client.[50] Self perceived empathy and emotional empathy were also explored.[51–53] Though professionals reacted positively to audio taping of the sessions.,[54] it is not widely used in training. Videotaping of the sessions involving the trainees is used. During clinical training, in psychotherapy, audio taping/videotaping of the therapy sessions are not mandatory and hence there is lack of adequate material to conduct research. Even as early as in 1974, videotapes were used in group psychotherapy sessions with male genital exhibitionists and the group dynamics is reported by an Indian researcher in USA.[16] We are yet to make use of the technology in clinical practice and research. The lack of research could be due to several factors: Time restraint, lack of facilities, inadequate supervised training and client’s perspectives about secrecy, privacy and stigma. However, the impact of therapist variables on patients has not been conducted so far.

### Where do we go from here?

There are very few studies on psychotherapy process or out come research in India. The reason for less number of research studies appearing in India may be a reflection of the priority of research in psychiatry in India. The course curriculum is one of the factors that could make a change in the scene. Psychotherapy is included in the curriculum of postgraduate training in psychiatry. However, there are no guidelines available at the national level on how and what skills need to be imparted. Introducing psychotherapy case submissions with adequate supervision as part of the curriculum is likely to generate the interest and improve the skills of the trainee psychiatrist.[27] Psychiatrists, who were trained through the curriculum wherein case submissions of psychological assessment were mandatory, had a better know-how of the relevance and usefulness of the psychological assessment tools. That probably led to the development as well as adaptation of new tools independently or in association with the fellow professionals. Psychotherapy supervision groups that have been found to be successful in some of the training centers may be started in other training centers too. Evidence-based psychotherapies are likely to be better accepted among the new entrants. Psychotherapy is moving towards integration of psychotherapies with divergent theoretical approaches. Unless there is exposure to different forms of psychotherapy, it may be difficult to integrate the concepts that are theoretically contradictory.

### The road ahead

Practicing psychotherapy is an interesting journey and supervision of psychotherapy, though emotionally taxing on the supervisor, is all the more interesting. Those who start off the journey hardly turn back, despite being pressurized with the demands of time and energy. All may not be willing to plunge into the journey. However, those who are willing may be facilitated to begin it with adequate and appropriate supervision. More number of trained psychiatrists who can strike a balance between the biological determinants of behavior and are willing to understand the psychopathology is likely to increase the number of research related to different approaches of psychotherapy. Indian philosophical psychology is a treasure to be unearthed in understanding the ‘person’ as well as in helping, but lacks empirical research evidence. Agarwal wrote two decades ago, “Psychotherapy which dominated psychiatry for long seems to have become relegated into oblivion”.[31] And through the journey of the IJP, so far, it is found to be true. Probably the new millennium might make the road smoother by publishing more research on evidence based psychotherapies that are suitable for the advantaged and the disadvantaged population of our country and thereby making a positive difference in the life of those who are suffering.
